# Comparative efficacy of aflibercept and ranibizumab in the treatment of age-related macular degeneration with retinal pigment epithelial detachment: a systematic review and network meta-analysis

**DOI:** 10.1186/s12886-023-03214-7

**Published:** 2023-11-21

**Authors:** Zuhua Sun, Yating Yang, Bing Lin, Ying Huang, Rong Zhou, Chun Yang, Yingzi Li, Shenghai Huang, Xiaoling Liu

**Affiliations:** 1 National Clinical Research Center for Ocular Diseases, Eye Hospital, Wenzhou Medical University, 270 Xueyuan Road, Wenzhou, 325027 Zhejiang Province China; 2 Yuncheng Central Hospital, Yuncheng City, 044000 Shanxi Province China

**Keywords:** Ranibizumab, Aflibercept, Anti-vascular endothelial growth factor, Age-related macular degeneration, Pigment epithelial detachment, Meta-analysis

## Abstract

**Objectives:**

To evaluate the efficacy of anti-vascular endothelial growth factor (VEGF) in treatment of age-related macular degeneration (AMD) with retinal pigment epithelial detachment (PED).

**Methods:**

Systematic review identifying studies comparing intravitreal ranibizumab (IVR), intravitreal aflibercept (IVA) and intravitreal conbercept (IVC) published before Mar 2022.

**Results:**

One randomized controlled trial and 6 observational studies were selected for meta-analysis (1,069 patients). The change of best corrected visual acuity (BCVA) in IVA 2.0 mg group was better than IVR 0.5 mg (average difference 0.07) and IVR 2.0 mg (average difference 0.10), the differences were statistically significant. The change of the height of PED in IVA 2.0 group was better than IVR 0.5 group (average difference 45.30), the difference was statistically significant. The proportion of patients without PED at last visit in IVA 2.0 group were better than those in IVR 2.0 group (hazard ratio 1.91), the difference was statistically significant. There was no significant difference compared with IVR 0.5 group (hazard ratio 1.45). IVA required fewer injections than IVR, with a mean difference of -1.58.

**Conclusions:**

IVA appears to be superior to IVR in improvement of BCVA, height decrease of PED and regression of PED with less injections in nAMD with PED.

**Supplementary Information:**

The online version contains supplementary material available at 10.1186/s12886-023-03214-7.

## Introduction

Age-related macular degeneration (AMD) is a leading cause of vision loss in the elderly population [1, 2]. The neovascular AMD (nAMD), also named as wet AMD, exudative AMD is responsible for 90% of acute blindness due to AMD [3]. Retinal pigment epithelial detachment (PED) is the separation of retinal pigment epithelium (RPE) from the Bruch’s membrane and is frequently seen in AMD [4, 5]. PED is present in about 30% to 80% of nAMD patients based on previous studies [6–9]. PEDs can be categorized as serous, hemorrhagic, drusenoid or fibrovascular according to their clinical appearance, optical coherence tomography (OCT) and fluorescein angiography (FA) findings [5, 10, 11]. Fibrovascular and serous PEDs are more commonly associated with nAMD [12]. PED is an important predictor of vision loss in AMD [8, 13, 14]. According to statistics, around half of the patients with a newly diagnosed PED will experience an average vision loss of > 3 lines over one year of follow-up [13].

The guidelines or expert consensus for AMD clinical diagnosis and treatment pathway recommend that the first-line treatment is intravitreal injection of anti-vascular endothelial growth factor (VEGF) agents [4, 15–18]. Anti-VEGF therapy can limit the progression of nAMD and stabilize or reverse vision loss [10]. The inter retinal fluid and subretinal fluid can decrease or disappear, but PEDs exist persistently after the anti-VEGF treatment reported in the PRONTO study [19]. At present, the mechanism of AMD complicated with PED is still unclear, the established hypothesis is that VEGF-associated fluid extravasation occurs from choroidal neovascularization (CNV) into the space between the RPE and Bruch’s membrane [5, 20]. Although the change of PED after the injection of aflibercept seemed superior to the other agents in the clinic by us, the clinical effect of anti-VEGF drugs in nAMD patients with PED is still lacking substantial evidence. So, we want to evaluate the efficacy of anti VEGF agents in the treatment of nAMD with PED, using a network meta-analysis. Currently, only ranibizumab, aflibercept and conbercept have been approved for nAMD in China. Therefore, these three agents were involved in this meta-analysis.

## Methods

This systematic review was conducted and reported in accordance with the Preferred Reporting Items for Systematic Reviews and Meta-Analyses (PRISMA) guideline for meta-analyses.

### Search strategy

Computer and manual searches were performed simultaneously by two searchers (CHR and GX). Computer study search was conducted using CNKI, Wanfang, VIP, China Biology Medicine disc, web of science, PubMed, Excerpta Medica data BASE (EMBASE (all via OVID Medline)), and the Cochrane Library from their inception until 4 Mar 2022. The manual searches were used to screen out studies that met the inclusion criteria from existing study references. The computer search and manual search processes were carried out independently by two searchers, and expert opinion was sought in the event of disagreement. Included trials are provided in Table 1.

**Table 1:** Basic characteristics of the studies included in the analysis

Study	Study Design	Country	Interventions and doses (mg)	Number of patients
Anagha 2018 [21]	Prospective study	Australia, Switzerland	IVA: 2.0 /IVR: 0.5	50/42
Sarraf 2016 [22]	Post-hoc analysis of RCT	U.S	IVR: 2.0 /IVR: 0.5	298/300
Chan 2015 [23]	RCT	U.S	IVR: 2.0 /IVR: 0.5	23/13
Au 2016 [24]	Retrospective study	U.S	IVA: 2.0 /IVR: 0.5	30/23
Park 2016 [25]	Retrospective study	South Korea	IVA: 2.0 /IVR: 0.5	74/87
Rouvas 2018 [26]	Retrospective study	Greece	IVA: 2.0 /IVR: 0.5	33/38
Ulusoy 2021 [27]	Retrospective study	Turkey	IVA: 2.0 /IVR: 0.5	25/33

*IVA* intravitreal aflibercept, *IVR* intravitreal ranibizumab

### Selection of studies

The search results were imported into the software of EndNote, and two independent researchers (CHR and GX) conducted two rounds of screening after excluding duplicated studies. Two researchers independently screened the studies based on the title and abstract information, reviewed the screening results with each other, and retained the studies that was agreed to be retained. For the controversial studies, a third senior independent researcher participated in the discussion and decision.

### Inclusion and exclusion criteria

#### Inclusion criteria

Criteria of study inclusion were defined as follows (PICOTS approach):

- (P) Patient was clinically diagnosed as PED secondary to AMD or polypoidal choroidal vasculopathy (PCV) or retinal angiomatous proliferation (RAP);
- (I) Interventions: intravitreal aflibercept, ranibizumab or conbercept;
- (C) Treatment comparisons: aflibercept, ranibizumab and conbercept;
- (O) Outcomes: any effect data reported (best corrected visual acuity (BCVA), height of PED, disappear of PED);
- (T) Minimal follow-up time: 6 months;
- (S) Studies were designed as randomized controlled trial (RCT), prospective studies, retrospective studies or real-world studies. Studies published in English or in Chinese were eligible for inclusion.

### Exclusion criteria

Studies with either of the following criterion were excluded:

1. CNV was secondary to some other diseases such as high myopia, angioid streaks, punctate inner choroidopathy, etc.;
2. Review articles (review papers, systematic review or meta-analysis);
3. Case report or case series; single-arm studies;
4. Dissertations, academic conferences, guidelines, editorials, letters, expert opinions, etc.;
5. Repeated published research;
6. Animal tests.

### Data extraction

The extracted data consists of three parts: (1) Study characteristics: including author, country, year of publication, study design, follow-up duration, and location of centers; (2) Characteristics of study patients and intervention measures: demographic characteristics of patients, sample size, disease information, drug in the experimental group and control group, etc.; (3) Outcomes: including BCVA, the height of PED and proportion of without PED. These data were independently extracted by two reviewers (TBY and PBZ). Any disagreement was referred to the third senior independent reviewer.

### Outcomes of interest

Primary outcomes were the (1) change of BCVA; (2) change of height of PED; (3) proportion of without PED at baseline and follow up; (4) numbers of injection.

### Methodological quality assessment of studies

The evidence quality of all included studies was evaluated by two reviewers using the GRADE assessment tool independently. In case of disagreements, group discussion and the third senior independent researcher were referred to solve it. In the GRADE approach, RCT was defined as high-quality evidence to support the estimates of intervention effects at the assessment, and observational studies were defined as low-quality evidence. In addition, there were five factors that could lead to the decline of evidence quality, including study limitations, imprecision, inconsistent results, indirect evidence, and possible publication bias. Three factors may improve the level of the evidence, including large effect size, the presence of dose–response effects and less of confounding factors. Ultimately, the corresponding quality of evidence for each outcome was assigned to one of four categories from high to very low [6].

### Heterogeneity analysis

The studies were analyzed for heterogeneity, including clinical, methodological and statistical heterogeneity. If obvious clinical heterogeneity or methodological heterogeneity was found among the studies, subgroup analysis will be directly performed until there was no obvious methodological or clinical heterogeneity among each subgroup.

### Statistical analysis

R statistical analysis software (R Studio Version 1.3.959) was used to perform hierarchical Bayesian analysis. The mean difference was used as the effect scale for the measurement data, Risk Ratio was used as the effect scale for the count data, the pooled point estimate and 95% confidence interval were calculated, respectively. All tests were two-sided, and *P* < 0.05 was considered statistically significant. The difference was statistically significant when neither the point estimate nor the confidence interval included the 0 value. Results of quantitative pair-wise analysis was presented using forest plots.

## Results

**Fig. 1:**
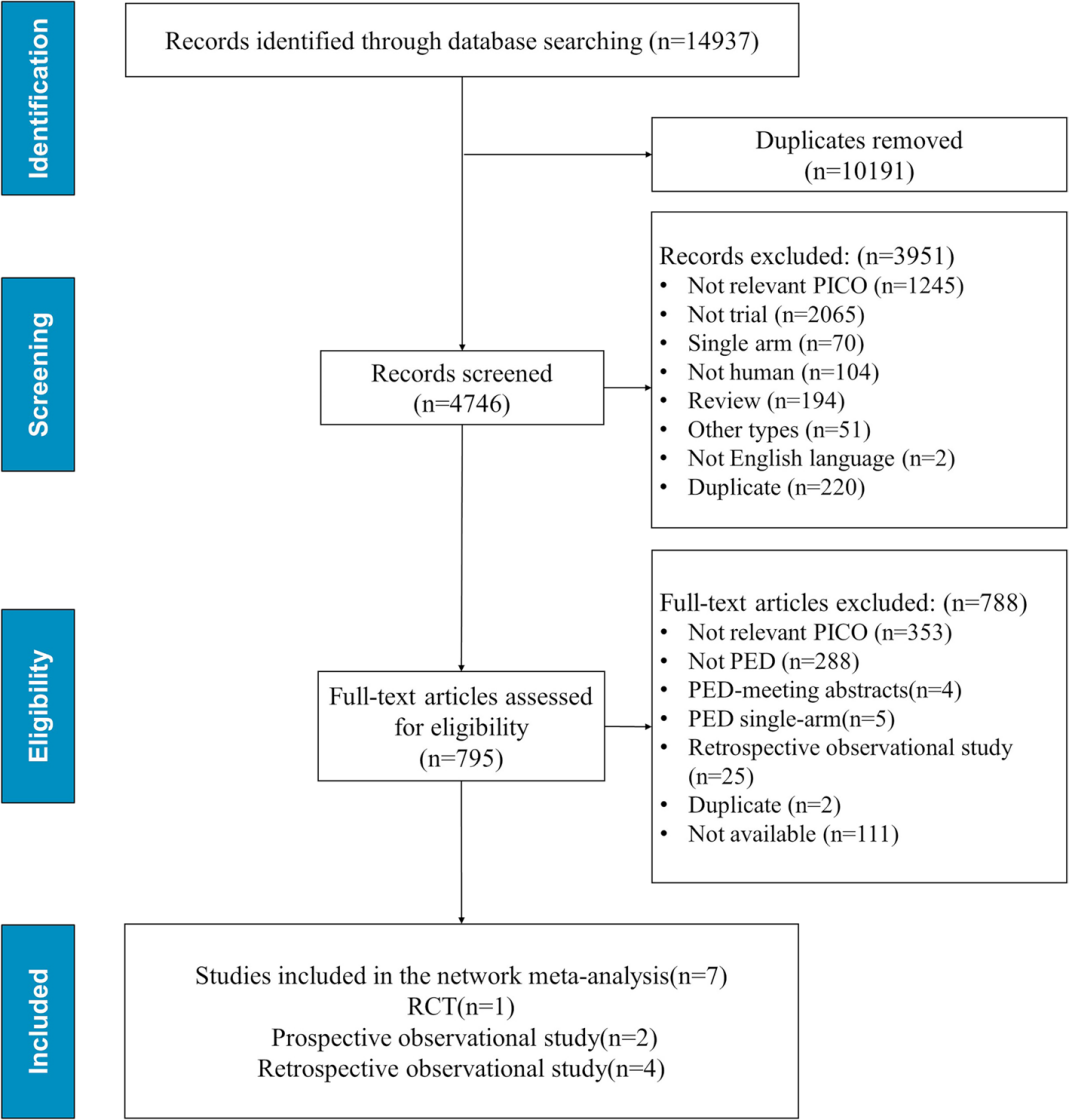
Study flow diagram Preferred Reporting Items for Systematic Reviews and Meta-Analyses (PRISMA) showing number of trials identified, included, excluded and reason for exclusion. RCT: randomized controlled trial; PICO: patient intervention control outcome; PED: pigment epithelium detachment

A total of 14,937 related studies were retrieved after a preliminary search. 10,191 duplicate articles were excluded, 3,951 articles were also excluded because of not meeting the requirements by reading the titles and abstracts, 795 articles were left. The full text of the remaining studies was re-screened, which did not meet the requirements was further excluded. Finally, 7 studies with a total of 1,069 patients were included. There was no direct or indirect comparison between aflibercept and conbercept in the PED patients that met the inclusion and exclusion criteria. Only the comparative studies between aflibercept and ranibizumab were retrieved. Study selection flow chart was shown in Fig. 1.

### Basic characteristics of included studies and quality assessment

Seven studies were finally included: 1 RCT, 2 prospective observational studies and 4 retrospective observational studies [21–27]. Basic characteristics of included studies were given in the Table 1.

#### Network structure

The network meta-analysis of this study belonged to the open-loop network. The intervention group received intravitreal injection of aflibercept (IVA) 2.0 mg monthly, while the control group received intravitreal injection of ranibizumab (IVR) 0.5 mg and 2.0 mg monthly, respectively. There was no direct comparison between IVA and IVR2.0.

#### Outcome indicator

The outcomes for analysis included the change of BCVA, the change of the height of PED from baseline and the proportion of patients without PED at baseline and during the follow-up. BCVA was measured according the Early Treatment Diabetic Retinopathy Study (ETDRS) chart or equivalent and recorded as LogMAR visual acuity. The height of PED was defined as the vertical distance from the outer line of RPE to the inner line of Bruch membrane on OCT. The follow up was 6 months or more. The data of each study was shown in Table 2.

**Table 2:** Change of best corrected visual acuity, height of PED and proportion without PED in each study

Study	Interventions (mg)	Follow-up (months)	Change of BCVA		Change of height of PED		Proportion without PED	
			**Average (LogMAR)**	**Standard deviation**	**Average (μm)**	**Standard deviation**	**Baseline**	**Last visit**
Anagha 2018 [21]	IVR 0.5	6	-0.13	0.04	-107.9	25.2	0/42	17/42
	IVA 2.0	6	-0.14	0.035	-69.9	23.93	0/50	14/50
Sarraf 2016 [22]	IVR 0.5	24	-0.18	0.014	-155.9	15.28	0/154	82/154
	IVR 2.0	24	-0.14	0.034	-191.1	13.9	0/158	111/158
Chan 2015 [23]	IVR 0.5	12	-0.11	0.122	-167.6	42.66	NA	NA
	IVR 2.0	12	-0.11	0.06	-245.06	32.22	NA	NA
Au 2016 [24]	IVA 2.0	12	0.03	0.108	-174.53	12.12	NA	NA
	IVR 0.5	12	0.1	0.058	-78.89	16.55	NA	NA
Park 2016 [25]	IVR 0.5	12	-0.19	0.041	-65.44	11.61	NA	NA
	IVA 2.0	12	-0.2	0.033	-84.81	12.21	NA	NA
Rouvas 2018 [26]	IVR 0.5	12	-0.03	0.032	-179.48	10.56	NA	NA
	IVA 2.0	12	-0.17	0.035	-226.51	9.51	NA	NA
Ulusoy 2021 [27]	IVR 0.5	12	-0.13	0.125	-45.75	16.14	NA	NA
	IVA 2.0	12	-0.21	0.084	-103.16	21.8	NA	NA

*IVA* intravitreal aflibercept, *IVR* intravitreal ranibizumab, *BCVA* best corrected visual acuity, *PED* pigment epithelial detachment

#### Quality assessment

The included studies were evaluated by GRADE, and the evaluation contents and results were referred to Supplementary Materials.

### Risk of bias assessment

Details on the risk of bias assessment are presented as Supplementary Materials.

### Efficacy analysis

#### Comparative analysis of BCVA

**Fig. 2:**
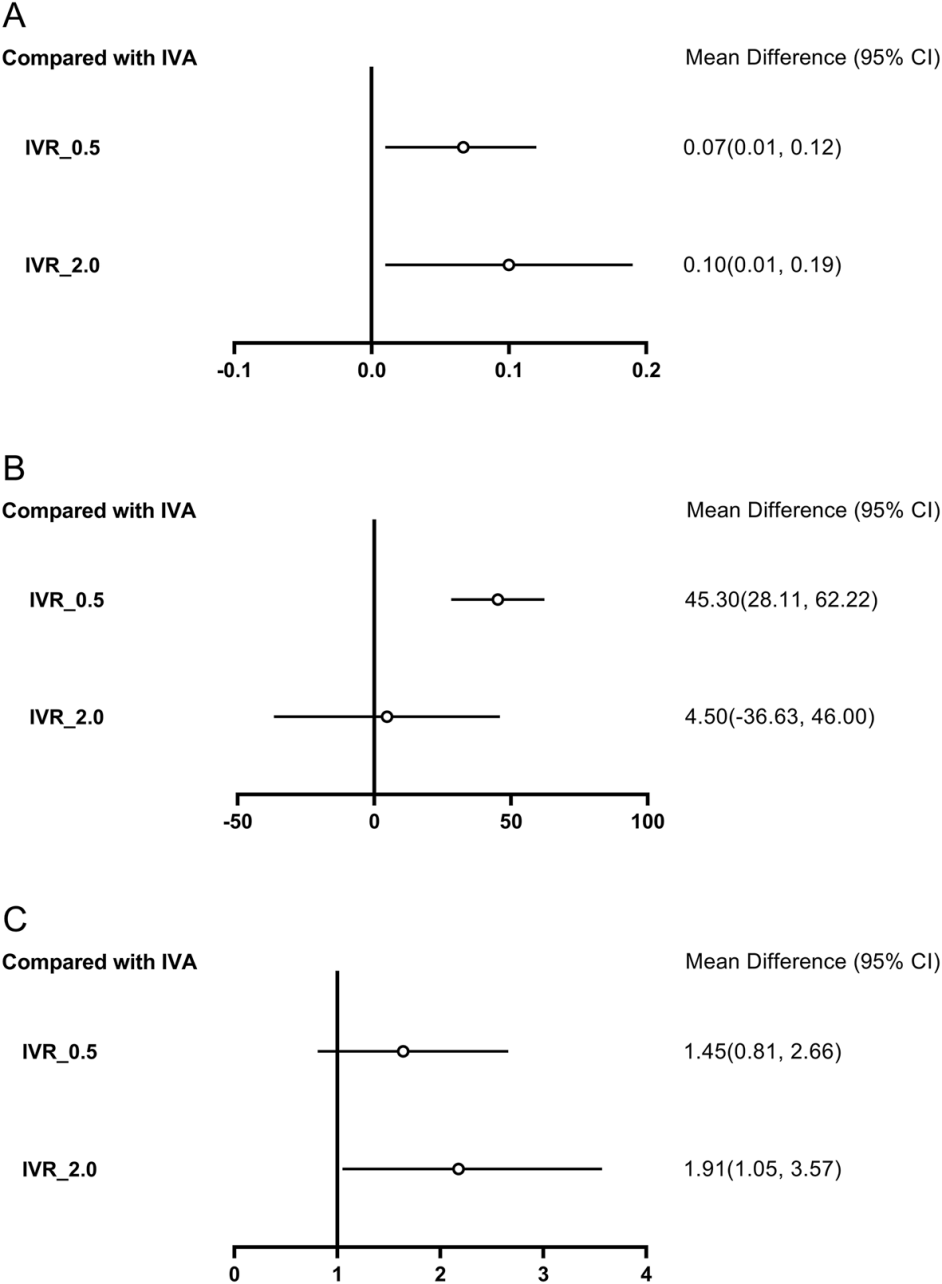
Forest plots. **A**. Change of BCVA between aflibercept and ranibizumab groups. **B**. Change of the height of PED between aflibercept and ranibizumab. **C**. The proportion of patients without PED at follow-up between aflibercept and ranibizumab. IVA: intravitreal aflibercept, IVR: intravitreal ranibizumab

BCVA was reported in seven studies. The change of BCVA before and after treatment was analyzed. The heterogeneity among studies was I^2^ = 8%, which was low. Comparing IVA with IVR 0.5, the difference was 0.07, 95%CI (0.01, 0.12). Comparing IVA with IVR 2.0, the difference was 0.10, 95%CI (0.01, 0.19). The change of BCVA in IVA 2.0 was better than IVR 0.5 and IVR 2.0, the difference was statistically significant (Fig. 2A).

#### Comparative analysis of the height of PED

The change of height of PED before and after treatment was analyzed. The heterogeneity among studies was I^2^ = 46%. Comparing IVA with IVR 0.5, the difference was 45.30, 95%CI (28.11, 62.22), comparing with IVR 2.0, the difference was 4.50, 95%CI (-36.63, 46.00). The change of the height of PED in IVA 2.0 was better than IVR 0.5. The difference was statistically significant. There was no statistical significance between IVA 2.0 and IVR 2.0 (Fig. 2B).

#### Comparative analysis of the proportion of patients without PED

Two studies reported the proportion of patients without PED. The heterogeneity between studies was I^2^ = 25%. Comparing IVR 0.5 with IVA 2.0, the hazard ratio was 1.45, with the 95% CI (0.81, 2.66); The hazard ratio of IVR 2.0 compared with IVA 2.0 was 1.91, with the 95% CI (1.05, 3.57). The changes in the proportion of patients without PED in IVA 2.0 were better than IVR 2.0. The difference was statistically significant. However, there was no significant difference compared with IVR 0.5 (Fig. 2C).

#### Comparative analysis of the number of injections

Included studies had different treatment frequency because of different treatment regimens. In this review, only three studies used IVA or IVR with a regimen of 3 + pro re nata (PRN), and one study used IVR 0.5 or 2.0 with a regimen of 4 + PRN or 12 injection monthly. One study used IVA or IVR without reporting the treatment regimen. During the 12 months follow-up, the mean number of injections for IVA and IVR were 7.29 ± 4.17 and 8.87 ± 3.92, respectively. Aflibercept required fewer injections than ranibizumab, with a mean difference of -1.58, with the 95% CI (-2.40, -0.76). There was significant difference between IVA and IVR (*t* = -3.78, *P* = 0.000).

## Discussion

The pathophysiology of PED with AMD is not well understood. Over expression of VEGF can lead to CNV. CNV could lead to the ingrowth of leaking vessels in the sub-RPE space through Bruch’s membrane, which may further lead to the formation of PED [5, 28]. The other proposed mechanisms include the abnormal vessels tend to leak and bleed because of lacking a proper inner blood–retina barrier and therefore, thus playing a central role in the formation of PED [5, 29, 30].

Regression of CNV can also bring about regression of PED during the anti-VEGF treatment. Many studies attempted to evaluate quantitative morphological parameters of PED on OCT in addition to studying the effects of its presence or absence. It is hoped that quantitative metrics of PED morphology may offer a more detailed characterization of PED evolution after treatment with anti-VEGF agents and allow the quantification of influence on visual outcomes. Previous studies reporting an association between PEDs and visual outcomes in patients with nAMD have yielded varying results with several studies concluding that the presence of PED does not impact vision whereas others show a negative correlation [6]. Parameters of PED that have been evaluated include height, width, greatest linear diameter, area, volume, and reflectivity of sub-RPE contents [6, 31]. The post hoc analysis of HAWK and HARRIER studies showed that greater PED thickness (≥ 200 μm) was associated with poorer visual outcomes and greater neovascular activity [32].The results about BCVA, PED height, PED regression rate and injection numbers were analyzed among the anti-VEGF agents in this meta-analysis.

In the change of BCVA, aflibercept was superior to ranibizumab 0.5 mg and 2.0 mg. A study has shown that IVA can lead to a significant reduction in PED height as well as to preserve BCVA stable over 1 year in the treatment of refractory vascular PED due to nAMD [33].

The post hoc analysis of HARBOR study analyzed the visual and anatomic outcomes of nAMD treated with ranibizumab at 24 months [22]. BCVA improvement in all groups were comparable in patients with or without PED at baseline. When analyzed by baseline PED height, BCVA improvement were comparable in all treatment groups at 24 months except for IVR 2.0 monthly in the extra-large PED group (PEDs ≥ 352 μm). At month 24, 53.2% (0.5 mg monthly), 44.5% (0.5 mg PRN), 70.4% (2.0 mg monthly), and 57.3% (2.0 mg PRN) of patients achieved complete regression of PED. In IVR 0.5 3 + PRN group, mean numbers of injections were similar for patients with PED present or absent at baseline (14.0 vs. 12.5). BCVA can be significantly improved regardless of PED absent or not, and the height of PED at baseline. There was no additional vision benefit with a higher dose of ranibizumab (2.0 mg).

For PED, there is currently no clear recommendation in the guideline consensus or substantial evidence showing the efficacy advantages of different drugs. The network meta-analysis showed that aflibercept was superior to ranibizumab in improving BCVA, reducing PED height and regressing the PED with less injections in patients with nAMD. The reason may be related to that aflibercept, a fusion protein of VEGF receptor-1 and VEGF receptor-2, can block all VEGF-A isoforms, VEGF-B and placental growth factor (PlGF) [34]. The molecular weight of aflibercept is longer than that of ranibizumab, and the dose is higher, which can let it penetrate all retinal layers, and effect longer period.

However, there are some limitations. Firstly, only three anti-VEGF agents most common being used currently in China were included. While there were few studies about conbercept on the treatment of nAMD with PED because the lack of approvement in other countries besides China. Secondly, there was lack of supporting data from a large number of clinical samples, so real-world research evidence was also included. There was lack of direct comparison between aflibercept and conbercept, as well as ranibizumab 2.0 mg. No analysis was made for different types of PED. Thirdly, the included studies have inconsistent confounding factors in terms of treatment time and number of treatments. In addition, PCV was not separated from nAMD although PED is more often happened in PCV.

## Conclusions

Based on available comparative studies, in terms of improvement in BCVA and decrease in height of PED, IVA appears to superior to IVR 0.5 in the treatment of nAMD with PED. Whereas IVA appears to have some benefits in terms of regression of PED as well as improvement in BCVA comparing with IVR 2.0. Meanwhile, the number of injections with IVA is less than with IVR. There was no direct or indirect comparison between aflibercept and conbercept in nAMD with PED meting the inclusion and exclusion criteria. Therefore, more studies about anti-VEGF agents in the treatment of nAMD with PED should be further evaluated.

### Supplementary Information

**Additional file 1: **Supplementary Materials. **Table S1.** Change of best corrected visual acuity quality assessment. **Table S2.** Change of the height of PED quality assessment. **Table S3.** Change of the proportion of patients without PED quality assessment.

## Data Availability

All data relevant to the study are included in the article or uploaded as supplementary information. The data for the meta-analyses conducted are included in the manuscript.

## Abbreviations

AMD: Age-related macular degeneration
BCVA: Best corrected visual acuity
CNV: Choroidal neovascularization
ETDRS: Early Treatment Diabetic Retinopathy Study
FA: Fluorescein angiography
IVR: Intravitreal ranibizumab
IVA: Intravitreal aflibercept
IVC: Intravitreal conbercept
OCT: Optical coherence tomography
PCV: Polypoidal choroidal vasculopathy
PED: Pigment epithelial detachment
PlGF: Placental growth factor
PRISMA: Preferred Reporting Items for Systematic Reviews and Meta-Analyses
PRN: Pro re nata
RAP: Retinal angiomatous proliferation
RCT: Randomized controlled trial
RPE: Retinal pigment epithelium
VEGF: Vascular endothelial growth factor
