# Supplementary material for: Comparative efficacy of aflibercept and ranibizumab in the treatment of age-related macular degeneration with retinal pigment epithelial detachment: a systematic review and network meta-analysis
Source: BMC Ophthalmol. 2023 Nov 21;23:473. doi: 10.1186/s12886-023-03214-7 (PMC10664577; doi:10.1186/s12886-023-03214-7)
Supplement: Supplementary file 1 — Additional file 1: Supplementary Materials. Table S1. Change of best corrected visual acuity quality assessment. Table S2. Change of the height of PED quality assessment. Table S3. Change of the proportion of patients without PED quality assessment. [file 12886_2023_3214_MOESM1_ESM.docx]

# **Supplementary Materials**

**1. Disease search terms**

| **disease** | **AMD** | **PED** | **CNV** | **PCV** | **RAP** |
| --- | --- | --- | --- | --- | --- |
| **English database search terms** | AMD | PED | CNV | PCV | RAP |
|  | ARMD | pigment* NEAR/2 epitheli* NEAR/2 detachment* | choroid* NEAR/2 neovascular* | polypoid* NEAR/2 choroid* NEAR/2 vasculopathy | retinal NEAR/2 angiomatous NEAR/2 prolifera* |
|  | maculopath* |  |  | polypoid* NEAR/2 choroid* NEAR/2 neovascular* |  |
|  | macul* NEAR/2 degeneration* |  |  | Branch* NEAR/2 vascular* NEAR/2 network* |  |
|  | macul* NEAR/2 dystroph* |  |  |  |  |
|  | macul* NEAR/2 atrophy* |  |  |  |  |
|  | | | | | |
| **Chinese database search terms** | macular degeneration | Pigment epithelial detachment | choroidal neovascularization | Polypoid choroidal vessels | retinal hemangioma-like hyperplasia |
|  | macular degeneration | PED | CNV | polypoid choroidal vessels | RAP |
|  | AMD |  |  | choroidal branching vascular network |  |
|  |  |  |  | PCV |  |

**2.Drug** **search terms**

| **drug** | **Aflibercept** | **Ranibizumab** | **Conbercept** |
| --- | --- | --- | --- |
| **English database search terms** | Aflibercept | Ranibizumab | Conbercept |
|  | eylea | Lucentis |  |
|  | VEGF Trap-Regeneron | lucentris |  |
|  | VEGF Trap | rhuFab V2 |  |
|  | VEGF Trap-Eye |  |  |
|  | vascular-endothelial-growth-factor-trap |  |  |
|  | | | |
| **Chinese database search terms** | Aflibercept | ranibizumab | Compaq |
|  | Apsipa | ranibizumab | Combusip |
|  | Alia | Novosti | Lang Mu |
|  | Seeming | Ranibizumab | Conbercept |
|  | Aflibercept | Lucentis |  |
|  | eylea |  |  |

**3. Comparison with previous searches (2020-11-25):**

|  | CNKI | Wanfang | VIP | CBM | Medline | Embase | WOS | Central |
| --- | --- | --- | --- | --- | --- | --- | --- | --- |
| quantity | 479 | 580 | 380 | 476 | 2425 | 3094 | 4043 | 1031 |
| After deduplication | 8492 | | | | | | | |

**4. As of the retrieval date 2022-03-04**

|  | CNKI | Wanfang | VIP | CBM | WOS | Embase | Cochrane | Medline-PubMed |
| --- | --- | --- | --- | --- | --- | --- | --- | --- |
| quantity | 854 | 855 | 275 | 1615 | 5032 | 1867 | 1099 | 3340 |
| Deduplication between databases (total) | 853 | 130 | 237 | 1007 | 5010 | 587 | 762 | 1458 |
| After deleting the same study as the old supplier study | 323 | 88 | 225 | 893 | 2269 | 168 | 190 | 590 |
| After deletion of documents identical to old supplier documents (total) | 4746 | | | | | | | |

**CNKI:** https://kns.cnki.net/

SU% = ("macular degeneration" + "macular degeneration" + "AMD" + "Pigment epithelial detachment" + "PED" + "choroidal neovascularization" + "CNV" + "Polypoid choroidal vessels" + "polypoid choroidal vessels" + "choroidal branching vascular network" + "PCV" + "retinal hemangioma-like hyperplasia" + "RAP") AND SU%=("Aflibercept" + "Apsipa" + "Alia" + "Seeming" + "Aflibercept" + "eylea" + "ranibizumab" + "ranibizumab" + "Novosti" + "Ranibizumab" + "Lucentis" + "Compaq" + "Combusip" + "Lang Mu" + "Conbercept") Chinese 854

**Wanfang:** http://new.wanfangdata.com.cn

theme:( "macular degeneration" or "macular degeneration" or "AMD" or "Pigment epithelial detachment" or "PED" or "choroidal neovascularization" or "CNV" or "Polypoid choroidal vessels" or "polypoid choroidal vessels" or "choroidal branching vascular network" or "PCV" or "retinal hemangioma-like hyperplasia" or "RAP") AND theme:("Aflibercept" or "Apsipa" or "Alia" or "Seeming" or "Aflibercept" or "eylea" or "ranibizumab" or "ranibizumab" or "Novosti" or "Ranibizumab" or "Lucentis" or "Compaq" or "Combusip" or "Lang Mu" or "Conbercept") Chinese study 855

**VIP:** [http://qikan.cqvip.com](http://qikan.cqvip.com/)

M= (macular degeneration OR macular degenerationOR AMD OR Pigment epithelial detachment OR PED OR choroidal neovascularization OR CNV OR Polypoid choroidal vessels OR polypoid choroidal vessels OR choroidal branching vascular network OR PCV OR retinal hemangioma-like hyperplasia OR RAP) AND M= (Aflibercept OR Apsipa OR Alia OR Seeming OR Aflibercept OR eylea OR ranibizumab OR ranibizumab OR Novosti OR Ranibizumab OR Lucentis OR Compaq OR Combusip OR Lang Mu OR Conbercept) 275

**CBM:** www.sinomed.ac.cn

1. "macular degeneration"[Common fields:intelligent] OR "macular degeneration"[Common fields:intelligent] OR "AMD"[Common fields:intelligent] OR "Pigment epithelial detachment"[Common fields:intelligent] OR "PED"[Common fields:intelligent] OR "choroidal neovascularization"[Common fields:intelligent] OR "CNV"[Common fields:intelligent] OR "Polypoid choroidal vessels"[Common fields:intelligent] OR "polypoid choroidal vessels"[Common fields:intelligent] OR "choroidal branching vascular network"[Common fields:intelligent] OR "PCV"[Common fields:intelligent] OR "retinal hemangioma-like hyperplasia"[Common fields:intelligent] OR "RAP"[Common fields:intelligent] Chinese study 14876
2. "Aflibercept"[Common fields:intelligent] OR "Apsipa"[Common fields:intelligent] OR "Alia"[Common fields:intelligent] OR "Seeming"[Common fields:intelligent] OR "Aflibercept"[Common fields:intelligent] OR "eylea"[Common fields:intelligent] OR "ranibizumab"[Common fields:intelligent] OR "ranibizumab"[Common fields:intelligent] OR "Novosti"[Common fields:intelligent] OR "Ranibizumab"[Common fields:intelligent] OR "Lucentis"[Common fields:intelligent] OR "Compaq"[Common fields:intelligent] OR "Combusip"[Common fields:intelligent] OR "Lang Mu"[Common fields:intelligent] OR "Conbercept"[Common fields:intelligent] Chinese study2728
3. #1 AND #2 -Chinese study1615

**WOS** https://www.webofknowledge.com/

#1 TS=(AMD OR ARMD OR maculopath* OR "macul* NEAR/2 degeneration*" OR "macul* NEAR/2 dystroph*" OR "macul* NEAR/2 atrophy*" OR PED OR "pigment* NEAR/2 epitheli* NEAR/2 detachment*" OR CNV OR "choroid* NEAR/2 neovascular*" OR PCV OR "polypoid* NEAR/2 choroid* NEAR/2 vasculopathy" OR "branch* NEAR/2 vascular* NEAR/2 network*" OR RAP OR "retinal NEAR/2 angiomatous NEAR /2 prolifera*") 104609

#2 TS=(Aflibercept OR eylea OR "VEGF Trap-Regeneron" OR "VEGF Trap" OR "VEGF Trap-Eye" OR "vascular-endothelial-growth-factor-trap" OR Ranibizumab OR Lucentis OR lucentris OR "rhuFab V2" OR "Conbercept") 15031

#3 #1 and #2 5032

**Embase** https://www.embase.com/

#1 AMD:ab,ti,kw OR ARMD:ab,ti,kw OR maculopath*:ab,ti,kw OR 'macul* NEAR/2 degeneration*':ab,ti,kw OR 'macul* NEAR/2 dystroph *':ab,ti,kw OR 'macul* NEAR/2 atrophy*':ab,ti,kw OR PED:ab,ti,kw OR 'pigment* NEAR/2 epitheli* NEAR/2 detachment*':ab, ti,kw OR CNV:ab,ti,kw OR 'choroid* NEAR/2 neovascular*':ab,ti,kw OR PCV:ab,ti,kw OR 'polypoid* NEAR/2 choroid* NEAR/2 vasculopathy': ab,ti,kw OR 'branch* NEAR/2 vascular* NEAR/2 network*':ab,ti,kw OR RAP:ab,ti,kw OR 'retinal NEAR/2 angiomatous NEAR/2 prolifera*':ab, ti,kw 65056

#2 Aflibercept:ab,ti,kw OR eylea:ab,ti,kw OR 'VEGF Trap-Regeneron':ab,ti,kw OR 'VEGF Trap':ab,ti,kw OR 'VEGF Trap-Eye':ab ,ti,kw OR 'vascular-endothelial-growth-factor-trap':ab,ti,kw OR Ranibizumab:ab,ti,kw OR Lucentis:ab,ti,kw OR lucentris:ab,ti,kw OR 'rhuFab V2 ':ab,ti,kw OR 'Conbercept':ab,ti,kw 10199

#3 #1 and #2 3270

#4 'human'/exp 24,478,816

#5 #3 and #4 3085

#6 'drug therapy': lnk 4,297,447

#7 #5 and #6 1867

**Cochrane library** https://www.cochranelibrary.com/

#1 (AMD OR ARMD OR maculopath* OR "macul* NEAR/2 degeneration*" OR "macul* NEAR/2 dystroph*" OR "macul* NEAR/2 atrophy*" OR PED OR "pigment* NEAR/2 epitheli* NEAR/2 detachment*" OR CNV OR "choroid* NEAR/2 neovascular*" OR PCV OR "polypoid* NEAR/2 choroid* NEAR/2 vasculopathy" OR "branch* NEAR/2 vascular* NEAR/2 network*" OR RAP OR " retinal NEAR/2 angiomatous NEAR/2 prolifera*"):ti,ab,kw 5310

#2 (Aflibercept OR eylea OR "VEGF Trap-Regeneron" OR "VEGF Trap" OR "VEGF Trap-Eye" OR "vascular-endothelial-growth-factor-trap" OR Ranibizumab OR Lucentis OR lucentris OR "rhuFab V2" OR "Conbercept"): ti, ab, kw 3018

#3 #1 AND #2 11099

**Pubmed** https://pubmed.ncbi.nlm.nih.gov/

***All Fields***

#1 "AMD" OR "ARMD" OR "maculopath*" OR "macul* degeneration*" OR "macul* dystroph*" OR "macul* atrophy*" OR "PED" OR "pigment* epitheli* detachment*" OR " CNV" OR "choroid* neovascular*" OR "PCV" OR "polypoid* choroid* vasculopathy" OR "branch* vascular* network*" OR "RAP" OR "retinal angiomatous prolifera*" [69,971](http://invalid.uri)

#2 "Aflibercept" OR "eylea" OR "VEGF Trap-Regeneron" OR "VEGF Trap" OR "VEGF Trap-Eye" OR "vascular-endothelial-growth-factor-trap" OR "Ranibizumab" OR "Lucentis" OR " lucentris" OR "rhuFab V2" OR "Conbercept" [7,556](http://invalid.uri)

#3 #1 and #2 3967

*Filters applied: MEDLINE.* 3340

|  |  |  | **Comment content** | | | | | | | | | **Number of cases** | | **Effect size** |
| --- | --- | --- | --- | --- | --- | --- | --- | --- | --- | --- | --- | --- | --- | --- |
| **Included studies** | **Study design** | **Comparative strategy** | **Risk of bias** | **Inconsistency** | **Indirect** | **Imprecision** | **Publication bias** | **Large effect value** | **Negative bias** | **Dose-response relationship** | **GRADE** | **Research group(n)** | **Control group(n)** | **MD (95%CI)** |
| **Change of BCVA** | | | | | | | | | | | | | | |
| **1 article** | **RCT** |  | Summarize all studies for judgment. | I^2 = 8%, no apparent inconsistency |  |  |  |  |  |  |  |  |  | 0.06589 (0.01151, 0.1203) |
| Chan 2015 |  | IVR 2.0 vs IVR 0.5 | Open-labe,unblinded, risk of selective reporting bias,treatment outcomes for each intervention group were not reported. | Not serious | Not serious | Not serious | Not serious | No | No | No | Middle | 13 | 23 |  |
| **6 articles** | **Observational study** |  | Summarize all studies for judgment. | Not serious | No major differences in populations, interventions, or measured outcomes; no indirect comparisons | The credible interval width is narrow enough (not sure how narrow it is) | Are the follow-up results complete? | RR>0.5 | The effect of confounding is to reduce the effect size | No |  |  |  |  |
| Anagha 2018 | Prospective study | IVA vs IVR 0.5 | No significant differences in population, intervention measures or measured outcomes, the follow-up results were relatively complete, the effect size was small, and there was no dose-effect relationship | Not serious | Not serious | Not serious | Not serious, no apparent publication bias | No | No | No | Low | 42 | 50 |  |
| Au 2016 | Retrospective study | IVA vs IVR 0.5 | Incomplete follow-up and possible publication bias. | Not serious | Not serious | Not serious | Serious limitations | No | No | No | Very low | 30 | 23 |  |
| Park 2016 | Retrospective study | IVA vs IVR 0.5 | No significant differences in population, intervention measures or measured outcomes, the follow-up results were relatively complete, the effect size was small, and there was no dose-effect relationship. | Not serious | Not serious | Not serious | Not serious, no apparent publication bias | No | No | No | Low | 74 | 87 |  |
| Sarraf 2016 | Post-hoc analysis of RCT | IVR 2.0 vs IVR 0.5 | No significant differences in populations, interventions, or measured outcomes, the follow-up results were relatively complete, and no PED type identification was performed in the included patients. | Not serious | Not serious | Not serious | Not serious, no apparent publication bias | more patients | No | No | Middle | 300 | 298 |  |
| Rouvas 2018 | Retrospective study | IVA vs IVR 0.5 | No significant differences in populations, interventions, or measured outcomes, and follow-up results were relatively complete. | Not serious | Not serious | Not serious | Not serious, no apparent publication bias | No | No | No | Low | 33 | 38 |  |
| Ulusoy 2021 | Retrospective study | IVA vs IVR 0.5 | No significant differences in population or measurement outcomes, follow-up results were relatively complete, and detailed doses of interventions were not specified. | Not serious | Not serious | Not serious | Not serious, no apparent publication bias | No | No | No | Low | 25 | 33 |  |
| **Change of height of PED** | | | | | | | | | | | | | | |
| **1 article** | **RCT** |  | Summarize all studies for judgment | I^2 = 46% Serious inconsistency |  |  |  |  |  |  |  |  |  | 45.3 (28.11, 62.22) |
| Chan 2015 | RCT | IVR 2.0 vs IVR 0.5 | Open-label, unblinded; risk of selective reporting bias, treatment outcomes for each intervention group were not reported. | Serious limitations | Not serious | Not serious | Not serious | No | No | No | Middle | 13 | 23 |  |
| **6 articles** | **observational study** |  | Summarize all studies for judgment | Serious limitations | No major differences in populations, interventions, or measured outcomes; no indirect comparisons. | The credible interval width is narrow enough (not sure how narrow it is) | Are the follow-up results complete? | RR>0.5 | The effect of confounding is to reduce the effect size | No |  |  |  |  |
| Anagha 2018 | Prospective study | IVA vs IVR 0.5 | The inconsistency with other studies was serious, no significant difference in population, intervention measures or measurement outcomes,relatively completely follow-up results, small effect size, and no dose-effect relationship | Serious limitations | Not serious | Not serious | Not serious | No | No | No | Low | 42 | 50 |  |
| Au2016 | Retrospective study | IVA vs IVR 0.5 | Incomplete follow-up and possible publication bias. | Serious limitations | Not serious | Not serious | Serious limitations | No | No | No | Very low | 30 | 23 |  |
| Park 2016 | Retrospective study | IVA vs IVR 0.5 | The inconsistency with other studies is serious, there is no significant difference in population, intervention measures or measurement outcomes, the follow-up results are relatively complete, the effect size is small, and there is no dose-effect relationship | Serious limitations | Not serious | Not serious | Not serious | No | No | No | Low | 74 | 87 |  |
| Sarraf 2016 | Post-hoc analysis of RCT | IVR 2.0 vs IVR 0.5 | No significant differences in populations, interventions, or measured outcomes. The follow-up results were relatively complete, and no PED type identification was performed. | Serious limitations | Not serious | Not serious | Not serious | More patients | No | No | Middle | 300 | 298 |  |
| Rouvas 2018 | Retrospective study | IVA vs IVR 0.5 | No significant differences in populations, interventions, or measured outcomes, and follow-up results are relatively complete. | Serious limitations | Not serious | Not serious | Not serious, no apparent publication bias | No | No | No | Low | 33 | 38 |  |
| Ulusoy 2021 | Retrospective study | IVA vs IVR 0.5 | No significant differences in population or measurement outcomes, follow-up results were relatively complete, and detailed doses of interventions were not specified. | Serious limitations | Not serious | Not serious | Not serious, no apparent publication bias | No | No | No | Low | 25 | 33 |  |
| **The proportion of patients without PED** | | | | | | | | | | | | | | |
| Anagha 2018 | Prospective study | IVA vs IVR 0.5 | The inconsistency with other studies was serious. No significant differences in population, intervention measures or measurement outcomes. The follow-up results were relatively complete. The effect size was not large, and there was no dose-effect relationship | Serious | Not serious | Not serious | Not serious | No | No | No | Low | 50 | 42 | 1.448 (0.811,2.6582) |
| Sarraf 2016 | Post-hoc analysis of RCT | IVR 2.0 vs IVR 0.5 | There were no major differences in populations, interventions, or measured outcomes, the follow-up results were relatively complete, and the included patients were not identified for PED type. | Serious | Not serious | Not serious | Not serious | Large number of patients | No | No | Middle | 158 | 154 | 1.910 (1.045,3.567) |

Table S1. Change of best corrected visual acuity quality assessment

| **Comparative measure** | **Risk of bias** | **Inconsistency** | **Indirect** | **Imprecision** | **Publication bias** | **Large effect value** | **Negative bias** | **Dose-response relationship** | **Mean Difference (95%CI)** | **Quality of evidence** |
| --- | --- | --- | --- | --- | --- | --- | --- | --- | --- | --- |
| IVA 2.0 mg vs. IVR 0.5 mg | Some | No | No | No | No | No | No | No | 0.0659 (0.0115, 0.120) | ++  Low |
| IVA 2.0 mg vs. IVR 2.0 mg | Some | No | Serious | No | No | No | No | No | 0.101 (0.0133, 0.189) | +  Very low |

Table S2. Change of the height of PED quality assessment

| **Comparative measure** | **Risk of bias** | **Inconsistency** | **Indirect** | **Imprecision** | **Publication bias** | **Large effect size** | **Negative bias** | **Dose-response relationship** | **Mean Difference (95%CI)** | **Quality of evidence** |
| --- | --- | --- | --- | --- | --- | --- | --- | --- | --- | --- |
| IVA 2.0 mg vs. IVR 0.5 mg | Some | Medium | No | No | No | No | No | No | 45.3 (28.1, 62.2) | ++  Low |
| IVA 2.0 mg vs. IVR 2.0 mg | Some | Medium | Serious | No | No | No | No | No | 4.5 (-36.6, 46.0) | +  Very low |

Table S3. Change of the proportion of patients without PED quality assessment

| **Comparative measure** | **Risk of bias** | **Inconsistency** | **Indirect** | **Imprecision** | **Publication bias** | **Large effect value** | **Risk ratio (95%CI）** | **Quality of evidence** |
| --- | --- | --- | --- | --- | --- | --- | --- | --- |
| IVA 2.0 mg vs. IVR 0.5 mg | Some | No | No | No |  | No | 1.441 (0.811, 2.658) | ++  Low |
| IVA 2.0 mg vs. IVR 2.0 mg | Some | No | Serious | No |  | No | 1.910 (1.045, 3.567) | +  Very low |
